# The Prevalence and Molecular Epidemiology of Hepatitis Delta Virus in Nigeria: The Results of a Nationwide Study

**DOI:** 10.3390/v16081236

**Published:** 2024-07-31

**Authors:** Ijeoma M. Ifeorah, Athenais Gerber, Samira Dziri, Solomon A. Bakarey, Frederic Le Gal, Gatuwa Aglavdawa, Chakib Alloui, Stephen O. Kalu, Peyou-Amed B. Ghapouen, Segolene Brichler, Olubusuyi M. Adewumi, Emmanuel Gordien

**Affiliations:** 1 Department of Medical Laboratory Science, Faculty of Health Sciences and Technology, University of Nigeria Enugu Campus, Enugu 200241, Nigeria; 2 Centre National de Référence des Hépatites B, C et Delta, Laboratoire de Microbiologie Clinique, Hôpital-Avicenne, Assistance Publique Hôpitaux de Paris, Université Sorbonne Paris Cité, 93000 Bobigny, France; athenais.gerber@aphp.fr (A.G.); samira.dziri@aphp.fr (S.D.); frederic.legal@aphp.fr (F.L.G.); chakib.alloui@aphp.fr (C.A.); segolene.brichler@aphp.fr (S.B.); 3 Institute for Advanced Medical Research and Training, College of Medicine, University of Ibadan, Ibadan 200212, Nigeria; drbakarey@yahoo.com; 4 Department of Hematology and Blood Transfusion, Federal Teaching Hospital Gombe, Gombe 620261, Nigeria; gatuwaharuna@gmail.com; 5 Medical Microbiology and Parasitology Department, Nnamdi Azikiwe University Teaching Hospital Nnewi, Nnewi 431101, Nigeria; kalu.stephen.o@gmail.com; 6 Department of Medical Laboratory Sciences, Taraba State University Jalingo, Jalingo 930283, Nigeria; peyou.amed@tsuniversity.edu.ng; 7 Department of Virology, Faculty of Basic Medical Sciences, College of Medicine, University of Ibadan, Ibadan 200212, Nigeria; adewumi1@hotmail.com

**Keywords:** HDV, genotype, Nigeria, nationwide study

## Abstract

Hepatitis delta virus (HDV) is a satellite of hepatitis B virus (HBV), which requires the HBV surface antigen (HBsAg) for its assembly and propagation. Although countries affected by HBV infection in Africa are well identified, data on HDV infection are still scarce, like in Nigeria, where HBV infection is endemic. In this study, we aimed to determine the prevalence of HDV infection and identify the circulating genotypes/strains in the country. A nationwide study was performed on 1281 HBsAg-positive samples collected from patients across eleven sites drawn from the six geopolitical zones in Nigeria. Anti-HDV antibody (HDV-Ab) screening and HDV-RNA viral load quantification were performed using a commercial ELISA assay and real-time RT-PCR kit, respectively. HDV genotyping was performed by the Sanger sequencing of amplicons from the so-called *R0* region of the viral genome, followed by phylogenetic analyses. Of the 1281 HBsAg-positive samples, 61 (4.8%) were HDV-Ab positive, among which, 12 (19.7%) were HDV-RNA positive. Genotypes were obtained for nine of them: seven “African” HDV-1, one “Asian/European” HDV-1 and one HDV-6. This study shows that Nigeria is a country of low HDV prevalence where mainly “African” genotype-1 strains are circulating.

## 1. Introduction

Hepatitis D virus (HDV) is a subviral satellite of Hepatitis B virus (HBV), requiring HBV envelope proteins for its assembly and propagation. Simultaneous infection with HBV and HDV (co-infection) or HDV infection in a chronically HBV-infected patient (super-infection) can occur. Globally, an estimated 12 million individuals (4.5%) among the general population of HBsAg carriers worldwide also have serological evidence of HDV infection with geographical heterogeneity [1]. So far, at the molecular level, eight HDV genotypes (HDV-1 to -8) and several sub-genotypes (within genotypes) have been described across the globe with distinct geographic origin and distribution [2,3,4]. While HDV-1 has been reported to have a wider geographical spread, others (HDV-2 to -8) showed a more confined distribution, with HDV-2 and -4 spreading in Asia, mainly in Eastern and Northern Asia; HDV-3 in South America; and HDV 5–8 seen majorly in sub-Saharan Africa (SSA) [3].

Nigeria, a country of over 200 million individuals with distinct ethnic populations spread across its six geopolitical zones, is a country of HBV hyperendemicity [5,6]. HBV prevalence varies greatly across these zones; however, a national average prevalence of 8.1% has been recorded among the adult population [5,7]. Available HDV reports showed prevalence ranging between 2 and 15% among different population groups in particular regions, but no data on some other regions [8,9,10,11,12,13,14]. Thus, HDV burden in many parts of Nigeria remains unknown, as screening for HDV in HBV-infected individuals (even among those with worsening liver disease due to HBV) is not a routine practice in most hospitals in Nigeria. Therefore, we carried out a nationwide screen of HDV from plasma samples obtained from 11 sites (including 10 states and the federal capital territory) spread across the six geopolitical zones of Nigeria.

## 2. Materials and Methods

### 2.1. Study Area

**Figure 1:**
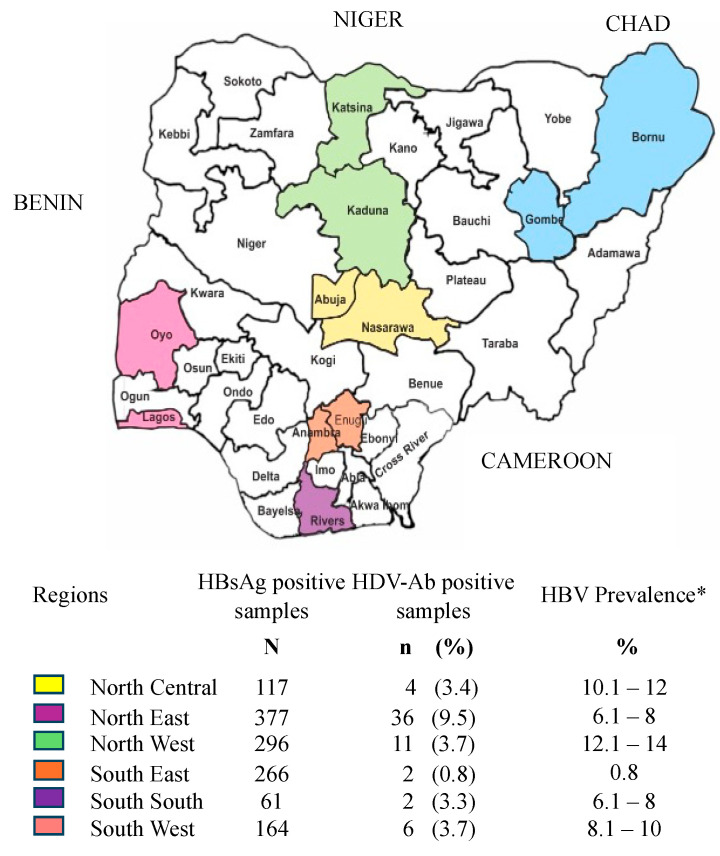
Prevalence of HDV-Ab in HBsAg-positive samples over the whole country. * HBV Prevalence as previously documented by Ajuwon et al. [5].

The study sites were hospitals selected from 11 sites (located in 10 states and the Federal Capital Territory) across the six geopolitical zones of Nigeria (Figure 1). The selected hospitals included major referral and/or tertiary hospitals that provide specialized health care services to the inhabitants of the city and nearby environs in the individual states where they are located.

### 2.2. Patients and Samples

A cross-sectional nationwide study was carried out among 1281 consenting chronic HBV patients attending the medical outpatient clinic (MOP) of the various selected health facilities between October 2017 and January 2019. The participants included 706 males and 575 females aged between 2 and 73 years (median age 34.6). The participants were asymptomatic HBsAg carriers who were referred to the specialist clinics on account of an initial positive HBsAg screen from either the general outpatient department (GOPD) within the same hospital or primary health care centers and other health care facilities around the major referral hospitals (study sites). Parental consent and assent (where applicable) were sought and obtained for underage participants before enrolment into the study. Consenting participants were rescreened onsite for HBsAg using a rapid HBsAg test kit (Acumen labs and diagnostic center, Bangalore, India) according to the manufacturer’s instructions. Thereafter, blood samples were collected by elbow venipuncture into EDTA tubes. Plasma was separated into appropriately labeled cryovials and stored at −20 degrees Celsius in the hospital laboratories. At the end of recruitment, the stored cryovials were sent to the Institute for Advanced Medical Research and Training (IAMRAT) University of Ibadan Nigeria where they were kept at −80 degrees Celsius until they were shipped to the French National Reference Laboratory for HDV (FNRL-HDV) for HDV antibody and RNA viral load quantification and genotyping.

### 2.3. HDV-Antibody, HDV-RNA Viral Load and HDV Genotyping

HDV-Ab positivity testing was performed using the LIAISON^®^ XL fully automated chemiluminescence analyzer from Diasorin (Saluggia, Italy), and HDV-RNA viral load was quantified in all positive samples using the CE marked Eurobio HDV kit EBX-004 (Courtaboeuf, Les Ulis, France) [15]. HDV genotyping was performed on all replicating strains using direct sequencing by the Sanger method of amplicons of the so-called *R0* region of the genome exactly as described earlier [3]. Phylogenetic trees were performed using well-characterized strains of all genotypes from our collection, including the ubiquitous HDV-1 from Asia, Europe and Africa.

## 3. Results

**Figure 2:**
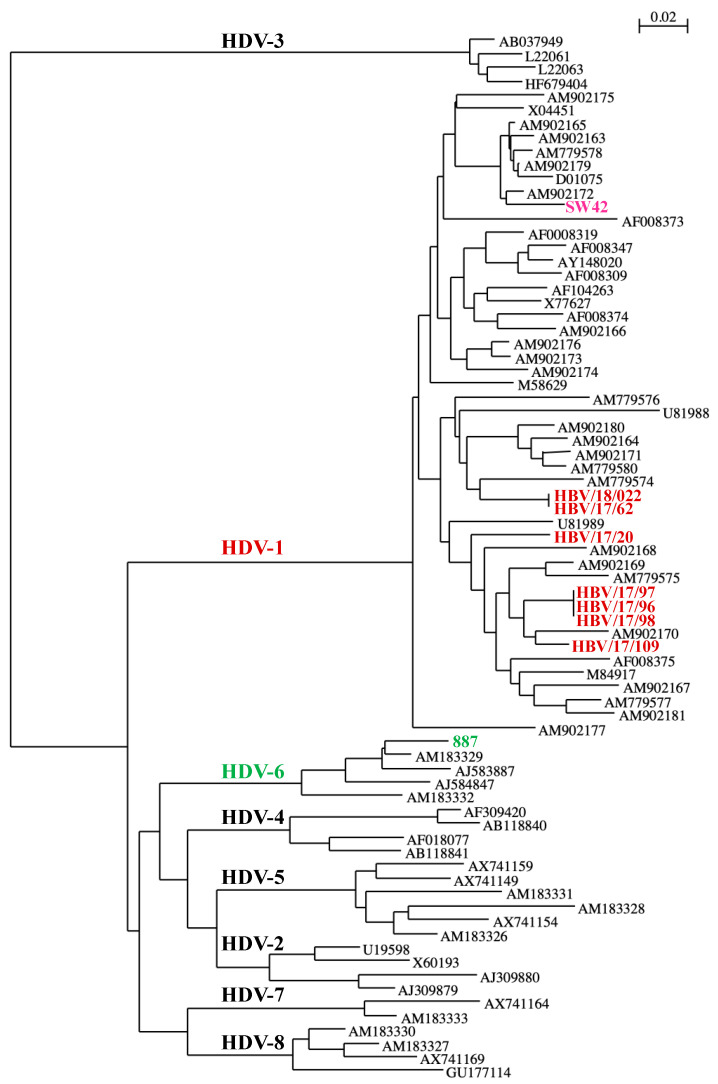
Phylogenetic tree showing genotype distribution of HDV strains from this study. Study isolates are indicated in red (African HDV-1), pink (non-African HDV-1) and green (HVD-6).

In total, 61 (4.8%) of the 1281 HBsAg-positive samples screened were found to be positive for anti-HDV antibodies (HDV-Ab). Nigeria can be classified as a low to very low country for HDV infection, ranging from 0.8 to 9.5%, with regional disparities. Indeed, a prevalence of nearly 10% was observed in the northeast region compared to 3 to 4% in other regions of the country (Figure 1). Only 12 patients (19.7%) were viremic, with a mean viral load of 4.4 log IU/mL, ranging from 2.8 to 7.4 log IU/mL. Due to the volume of samples available, genotypes could only be obtained from nine of the twelve viremic patients, among which, seven were the African HDV-1 genotype; one a non-African (European/Asian) HDV-1 {(sample SW42)-possessing alanine instead of serine at amino acid position 202 of the large delta protein and the remainder were HDV-6 (Figure 2). Of note, three HDV-1-strains infecting patients who were siblings were identical, indicating an intrafamilial transmission of the infection.

## 4. Discussion

The pooled anti-HDV prevalence of 4.8% from this study suggests that Nigeria is a country of low endemicity for HDV infection among individuals with asymptomatic chronic HBV infection, albeit with regional variations. Similar observations have been noted in previous studies, with reported prevalence ranging from 0 to 5.6% in similar cohorts [9,11,16,17,18]. However, higher anti-HDV prevalences of 12.5% and 16.7% have also been reported in similar cohorts in Nigeria [19,20]. This observed difference in rates reported by these other studies is probably due to variations in sample size, notable regional variations in HDV prevalence, assay variation or other factors.

In addition, the prevalence of viremic patients was low, as only 19.7% of the infected patients actively replicated the virus even while they were not on any treatment for HDV. This low HDV viremia among HDV-infected persons in sub-Saharan Africa (SSA) has been noted by Stockdale et al. [10] in a systematic review and meta- analysis describing the global prevalence of hepatitis Delta infection. Indeed, other studies have documented low HDV RNA among HDV-Ab-positive samples [8,9,21]. Factors that may be responsible for the low level of HDV active replication in the SSA population are still not clear. However, one could hypothesize earlier infection at birth or in the perinatal period with the immune tolerance phenomenon. Regional disparities, especially in the northeast, are seen and are very likely related to the common border with Cameroon, a country of high HDV endemicity. As expected, African genotypes, mainly HDV-1, are spreading in the country. However, the main results of this remarkable nationwide study will need to be consolidated with larger sampling because of the low prevalence of HDV infection. In this setting, the HDV-Ab reflex testing of all HBV-infected patients should be implemented. The development of the newly described rapid diagnostic test [22] would be of great interest.

## Data Availability

The raw data supporting the conclusions of this article will be made available by the authors on request.
